# Morphometric Analysis of the Intestine in Experimental Coccidiosis in Broilers Treated with Anticoccidial Drugs

**Published:** 2018

**Authors:** Sedigheh NABIAN, Fatemeh ARABKHAZAELI, Parvaneh SEIFOURI, Alireza FARAHANI

**Affiliations:** 1. Dept. of Parasitology, Faculty of Veterinary Medicine, University of Tehran, Iran; 2. Dept. of Clinical Sciences, Faculty of Veterinary Medicine, University of Tehran, Iran

**Keywords:** Anticoccidal drug, Experimental coccidiosis, Histomorphometric analysis, Intestinal macroscopic lesions, *Eimeria*

## Abstract

**Background:**

Coccidiosis causes morphologic alteration in intestinal mucosa resulting in reduction of absorptive surface. Anticoccidials used as feed additives may induce changes in the intestinal mucosa. This study was designed to assess intestinal morphometry in broilers infected with *Eimeria* under different anticoccidial treatments.

**Methods:**

To evaluate the effect of salinomycin and amprolium+ethopabate on intestinal morphometry in broilers experimental coccidiosis, in Tehran, Iran in May 2015, fifty-four Ross 308 birds were randomly divided into two challenged and unchallenged groups at the age of 12 days. The birds were challenged with *Eimeria* field isolate at day 14. Different growth and parasitological parameters including weight gain, feed consumption, FCR, macroscopic lesion score and oocyst score were recorded 7 d post-inoculation. Histological sections from four main parts of intestine (anterior, middle, lower intestines and cecum) were prepared and analyzed. Villus width and length and total mucosal thickness were measured microscopically.

**Results:**

Amprolium+ethopabate and salinomycin significantly reduced coccidiosis gross lesions in infected birds. Microscopically anticoccidial administration in the presence of infection has significantly increased the villus length while the presence of amprolium+ethopabate in the absence of infection has greatly increased the mucosal thickness and villi height in comparison to the control group.

**Conclusion:**

Anticoccidials may induce some histological changes in the mucosa when there is no parasite to be affected. Some of these effects may be advantageous for the intestinal epithelium integrity and hence the birds’ performance.

## Introduction

*Eimeria,* an apicomplexan protozoan parasite, is the cause of coccidiosis in the poultry industry. Six species of *Eimeria* are known to have an adverse economic impact on chicken (1) and annually impose nearly 2 billion US$ lose on commercial chicken producers at least 1.5 (2, 3). The integrity of the gastrointestinal (GI) tract is definite in digestion and absorption of nutrients and hence in the gainful poultry production (4). *Eimeria* proliferates in all parts of the intestinal tract, destructs the epithelial cells and thus disrupts nutrient absorption, weight gain and therefore financial effectiveness (4). The capability of the intestinal tract is adjustable based on different stimuli such as therapeutic or herbal feed additives and even pathogenic microorganisms. Anticoccidial food additives are the major route of coccidiosis prevention in today’s intensive broiler production industry. Amprolium occludes thiamin transporters in *Eimeria* species and salinomycin is an ionophore anticoccidial drug which alters the parasites’ cation balance (5, 6). Few studies have investigated the effect of anticoccidials on intestinal histomorphometry during a coccidial challenge.

We evaluated the influence of amprolium+ ethopabate and salinomycin on macroscopic intestinal lesions and microscopic intestinal morphology of infected broilers in experimental coccidiosis.

## Materials and Methods

### Oocyst collection

Litters of a commercial broiler house in west of Iran, Hamadan, was inspected for *Eimeria* oocysts (7, 8). Isolated oocysts were purified and identified (based on shape index) as *E. acervulina, E. maxima, E. tenella*, and *E. necatrix*. The challenge dose was chosen to induce the most pathologic lesions and the least mortality based on the experiment performed on four 14 d old chicks (9).

### Animals and experiment design

In Tehran, Iran in May 2015, 41 one-day-old male broilers (Ross 308) were randomly allocated to 6 groups each included three replicates of 3. The experiment was conducted in battery cages under controlled condition. The negative control, drug control, and treatment groups are summarized in Table 1.

**Table 1:** Experimental design for evaluation of the effect of salinomycin and amprolium+ethopabate on broilers intestinal morphometry

***Groups***	***Inoculated infective dose***	***Anticoccidials***
1	250000 oocyst/bird	Salinomycin @ 500 ppm
2	250000 oocyst/bird	Amprolium+ethopabate @ 500 ppm
3	250000 oocyst/bird	Non-medicated
4 (salinomycin control)	-	Salinomycin
5 (amprolium+ethopabate control)	-	Amprolium+ethopabate
6 (Negative control)	-	Non-medicated

The diet was formulated to meet all essential nutrients for the birds, and food and water were provided *ad-libitum*. No vaccine was administered during the experiment. To enable individual data recording, tags were attached to the birds’ leg.

At day 12^th^, anticoccidials were introduced in the birds’ diets and the diet was provided up to 7 d post-inoculation. Kimiamycin® 12 (Kimiafaam Group, Iran) and Ethoamprox® (JamedatAfagh Pharmaceutical Company, Iran) were prescribed 500 ppm in the feed. Overall, 14-day-old birds were challenged orally. On the day of experimental challenge and a week later, all of the birds were weighed individually. Weight gain (WG) and feed intake (FI) were recorded. Gross lesion score (GLS), microscopic lesion score (MLS) and oocysts index (OI) were investigated after necropsy and feed conversion ratio (FCR) was calculated consequently (8, 10).

### Pathological assay

At the end of the experiment (21st day), all the birds were euthanized by neck severing and necropsied. For the preparation of pathologic samples the whole GI tract was removed, slit open and both the mucosal surface and the unopened serosal surface of the intestine were examined for macroscopic lesions (11). Fragments of about two centimeters from the upper intestine (duodenum and jejunum), mid intestine, lower intestine (ileum) and cecum were cut, pinned and stowed in 10% buffered formalin solution. The segments were processed by routine histology methods, embedded longitudinally in paraffin, sectioned 5 μm thick parallel to the cut edge (Microm, Germany) and stained with hematoxylin and eosin (H&E) for microscopic evaluations (Nikon Eclipse E400, USA). At least 6 histological sections for each bird, totalizing about 300 slides were prepared and analyzed using H&E staining.

Microscopic lesion score was assessed by evaluating villus height, total mucosal thickness, the severity of villus infection and the distribution scores (12). Villus height was determined by measuring the length of 10 intact villi, from the crypt mouth to the apical villus region. Tip of villus to the base of the crypt was the criteria for total mucosal thickness. The percentage of the parasitized villi in four microscopic fields was used for determination of the severity of villus infection. The presence of parasite stage with ×10 magnification in four microscopic fields was used for establishing the distribution score (12).

### Statistical analysis

ANOVA, two-way *t*-test and Tukey’s multiple range tests were used to determine the significance of differences among groups (SPSS 15.0, Chicago, IL, USA) at *P*≤0.05.

## Results

### Growth performance

All of the unchallenged groups including treated and untreated had the highest average weight gain (352–371g) while the lowest level (148 g) was recorded for the untreated challenge group (*P*<0.05). The average weight gain of challenged groups either treated with salinomycin or amprolium+ ethopabate was significantly lower than unchallenged groups. However, salinomycin was more efficient weight gain in the experimental challenge. The same results were obtained for feed conversion ratio (Table 2).

**Table 2:** Evaluation of intestinal morphometry in chicken with experimental coccidiosis receiving dietary anticoccidials

***Experimental group***			***Average weight gain (g)***	***Oocyst index***	***Feed conversion ratio (g/g)***	***Gross Lesion score***	**Villus height (μm)**	**Total mucosal thickness (μm)**	**Severity of villus infection**	**Distribution score**
1	*Eimeria* mix sp. isolate	Salinomycin	230.5^b^	2^b^	1.74^c^	1.5^b^	556^a^	884^a^	1.89^a^	1.7^a^
2	*Eimeria* mix sp. isolate	Amprolium + ethopabate	172.7^c^	2.1^b^	2.1^b^	2.1^b^	531^a^	856^b^	2.16^a^	1.97^a^
3	*Eimeria* mix sp. isolate	Untreated	148^d^	2.6^a^	2.75^a^	2.3^a^	456^b^	822^c^	1.9^a^	1.7^a^
4	unchallenged	Salinomycin	368^a^	0	1.55^c^	-	498^b^	833^c^	0	0
5	unchallenged	Amprolium+ethopabate	352^a^	0	1.58^c^	-	552^a^	874^a^	0	0
6	unchallenged	Untreated	371^a^	-	1.65^c^	-	477^b^	808^d^	0	0

a–d Means sharing the same superscripts within each section do not differ (*P*≤0.05).

### Oocyst scores and gross lesions

As it was expected no oocyst was observed in unchallenged groups, while the oocyst index in challenged untreated group was significantly higher than challenged treated groups (*P*<0.05). The challenged untreated group had the highest gross lesion score (2.3) and the lowest GLS was determined for birds challenged-treated with amprolium+ethopabate (2.1) and challenged-treated with salinomycin (1.5), respectively. The intestinal gross examination of birds showed varying degrees of lesions in different parts of the intestines. These differences were significant between treated and untreated challenged groups. Gross lesions were varying from few scattered petechiae, ladder-like necrosis lesions to ballooned intestine, severe hemorrhage, and cecal cord.

### Intestinal histomorphometry

In this study, histopathological findings including villus height, total mucosal thickness, the severity of villus infection, and distribution score were measured. The distribution score and the severity of villus infection in different groups were between 1.7–1.97 and 1.9–2.16 respectively, which the highest value for both indexes belonged to amprolium+ethopabate treated groups.

No differences were observed in distribution score and the severity of villus infection among different treatments while the total mucosal thickness showed significant differences between different groups. Comparing the total mucosal thickness in different groups reveals better results in the two treated groups in comparison with the control group. The highest mucosal thickness was recorded for the challenged birds receiving salinomycin and unchallenged birds treated with ethoamprox. The lowest mucosal thickness was seen in the control group (unchallenged-untreated birds).

The villus height in birds challenged and treated with both of drugs and unchallenged birds treated with ethoamprox were significantly higher than the control group, the unchallenged birds treated with kimiamycin and the challenged-untreated birds, while no difference was observed between the two treated groups. There was a significant difference in villus height of unchallenged birds treated with ethoamprox (552μm) comparing with unchallenged either untreated or treated birds with kimiamycin (498 μm) (Table 2).

### Histopathologic alterations

**Fig. 1:**
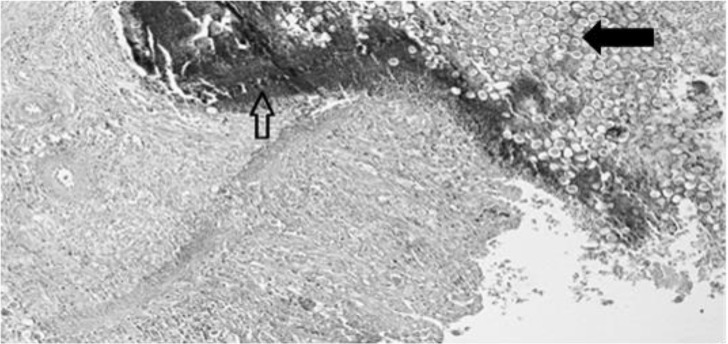
Histopathological section (H&E staining) (200×). Hemorrhagic enteritis, diffuse infiltration, severally expanding lamina propria (hollow arrow) and numerous coccidian parasites at various stage of development (filled arrow) are visible

In challenged untreated groups small intestine histopathological observation included diffusely infiltrating and severely expanding lamina propria with marked mixed inflammatory cell infiltrate mostly composed of lymphoblasts, with fewer histiocytes, plasma cells, and eosinophils. This inflammatory reaction extended multifocally and transmurally with mild accumulations on the serosal surface. Within the luminal epithelial cells, coccidian parasites at various stages of development were obvious. Abundant macrogamonts characterized by a peripheral ring of large eosinophilic granules, less abundantly microgamonts and schizonts were seen. Tip of villi was mostly broken off leading to truncation and fusion of villi and thickening of the mucosa. In some cases, severe damage of mucosa leading to hemorrhagic enteritis were noted (Fig. 1).

## Discussion

Infection with *Eimeria* causes morphologic alteration in intestinal mucosa resulting in the reduction of absorptive surface. Various methods including gross and microscopic lesion scoring can be applied to evaluate the severity of coccidiosis, different drug’s efficacy rate and routine coccidiosis screening in broiler flocks (13, 14). We tried to assay the intestinal morphometric alteration in experimental coccidiosis in broilers treated with salinomycin and amprolium+ ethopabate.

Administration of both drugs improved growth performance, gross lesion score and different microscopic lesions measured in this study, in comparison to challenged untreated group. No significant differences in the average weight gain and FCR were noted between unchallenged birds treated with either drug with the control group. Salinomycin had a better effect on average weight gain and feed conversion ratio. These results are corresponding to the findings (15), that reported amprolium+ethopabate has a good effect in mean body weight, FCR, villus height and mucosal thickness and (16), salinomycin could induced growth promotor effects and improve feed conversion and body weight gain via changing the composition and activities of gut microflora. A study on the effect of four anti-coccidial drugs on the normal chicken intestinal morphology concluded that anticoccidial drugs diclazuril, semduramicin, salinomycin, and maduramicin in the absence of coccidiosis had adverse effects on chicken performance and intestinal morphology (17). Likewise, salinomycin may suppress weight gain and FCR in the absence of coccidiosis (18), in this experiment unchallenged birds receiving salinomycin had average weight gain and FCR comparable to the negative control group.

Macroscopically amprolium+ethopabate and salinomycin significantly reduced coccidiosis gross lesions in challenged birds comparing to untreated birds. Microscopically anticoccidial administration in the presence of infection has significantly increased the villus length of the intestinal mucosa. Long villi and shallow crypts are essential for normal nutrient absorption in birds. Additionally, the presence of amprolium+ethopabate in unchallenged birds has greatly increased the mucosal thickness and villi height in comparison to the control group. Increase of villus height in the unchallenged group treated with amprolium+ethopabate was due to direct drug effect (19), about diclazuril. Regarding salinomycin in unchallenged birds, the total mucosal thickness was increased but the villus height was not significantly influenced. The severity and distribution scores, indicative of parasite reproduction, were not affected by anticoccidials used in this study. Gross lesion alone can underestimate *Eimeria* infection within broiler flocks (20); hence checking microscopic lesions may be used complementarily for monitoring coccidiosis and efficacy of different control measures implied (6). Microscopy has added benefits to gross lesion scoring as it readily detects not only oocysts but also developmental stages of the parasite (5).

In a study designed to assess the potential impact of litter fermentation and diclazuril administration on intestinal macroscopic and microscopic lesions caused by E. acervulina, both measurements reduced villus atrophy and induced greater absorptive area (19). Villus cell proliferation or apoptosis is an adaptive reaction to alterations imposed by different stimuli or pathogens (21). Broilers with dietary salinomycin had vaguely enhanced FCR but reduced villus height (22). With *Eimeria* proliferating in the mucosa, the effect of anticoccidials can be attributable to their effect on the parasite life cycle (18, 19) while with the absence of parasite the drugs may induce different host interaction in the intestinal mucosa. Feed components, pathogens and etc. interact with the normal chicken growth rate by modifying proliferation and maturation of cells in the small intestinal mucosa (21, 23). As anticoccidials are used as feed additives and can influence the intestinal cell proliferation studying the effects of these compounds in the presence and absence of *Eimeria* infection may lead to a better therapeutic outcome.

## Conclusion

Microscopic lesions, as gross lesions, in the intestinal mucosa of broilers infected with *Eimeria* sp. is directly influenced by anticoccidial feed additives. The anticoccidial can induce some histological changes in the mucosa when there is no parasite to be affected. Some of these outcomes may be advantageous for the integrity of the intestinal epithelium and hence the birds’ production performance.
